# Evaluation of a universal coverage bed net distribution campaign in four districts in Sofala Province, Mozambique

**DOI:** 10.1186/1475-2875-13-427

**Published:** 2014-11-05

**Authors:** Mateusz M Plucinski, Silvia Chicuecue, Eusébio Macete, James Colborn, Steven S Yoon, S Patrick Kachur, Pedro Aide, Pedro Alonso, Caterina Guinovart, Juliette Morgan

**Affiliations:** Division of Parasitic Diseases and Malaria, Center for Global Health, Centers for Disease Control and Prevention, Atlanta, GA 30333 USA; Epidemic Intelligence Service, Centers for Disease Control and Prevention, Atlanta, GA 30333 USA; Manhiça Health Research Centre, Manhiça, Mozambique; Direcção Nacional de Saúde, Ministério da Saúde, Maputo, Moçambique; Instituto Nacional de Saúde, Ministério da Saúde, Maputo, Moçambique; Barcelona Centre for International Health Research (CRESIB), Hospital Clínic-Universitat de Barcelona, Barcelona, Spain

**Keywords:** Long-lasting insecticidal net, Malaria, *Plasmodium falciparum*

## Abstract

**Background:**

Malaria is the leading cause of death in Mozambique in children under five years old. In 2009, Mozambique developed a novel bed net distribution model to increase coverage, based on assumptions about sleeping patterns. The coverage and impact of a bed net distribution campaign using this model in four districts in Sofala Province, Mozambique was evaluated.

**Methods:**

Paired household, cross-sectional surveys were conducted one month after the 2010 distribution of 140,000 bed nets and again 14 months after the campaign in 2011. During household visits, malaria blood smears were performed and haemoglobin levels were assessed on children under five and data on bed net ownership, access and use were collected; these indicators were analysed at individual, household and community levels. Logistic regression was used to evaluate predictors of malaria infection and anaemia.

**Results:**

The campaign reached 98% (95% CI: 97-99%) of households registered during the precampaign listing, with 81% (95% CI: 77-85%) of sleeping spaces covered by campaign bed nets and 85% (95% CI: 81-88%) of the population sleeping in a sleeping space with a campaign bed net designated for the sleeping space. One year after the campaign, 65% (95% CI: 57-72%) of sleeping spaces were observed to have hanging bed nets. The proportion of sleeping spaces for which bed nets were reported used four or more times per week was 65% (95% CI: 56-74%) in the wet season and 60% (95% CI: 52-68%) in the dry season. Malaria parasitaemia prevalence in children under five years old was 47% (95% CI: 40-54%) in 2010 and 36% (95% CI: 27-45%) in 2011. Individual-level malaria infection and anaemia were significantly associated with community-level use of bed nets.

**Conclusions:**

The campaign using the novel distribution model achieved high coverage, although usage was not uniformly high. A significant decrease in malaria parasitaemia prevalence a year after the campaign was not observed, but community-level use of bed nets was significantly associated with a reduced risk for malaria infection and anaemia in children under five.

**Electronic supplementary material:**

The online version of this article (doi:10.1186/1475-2875-13-427) contains supplementary material, which is available to authorized users.

## Background

Malaria is a leading cause of mortality in Mozambique, with 35% of children aged under five years testing positive for malaria parasites in 2011 [1]. Malaria control efforts in Mozambique include insecticide-treated bed net distribution, indoor residual spraying, and improving access to timely and accurate malaria diagnosis and artemisinin-based combination therapy among other priority malaria control interventions.

The national vector control strategy includes achieving universal access to at least one prevention strategy, with every individual either living in a household sprayed with insecticide or having access to a long-lasting insecticidal net (LLIN). Insecticide-treated bed nets are known to decrease all-cause child mortality (by 22%) and malaria morbidity [2], and are also able to measurably decrease community-wide malaria transmission [3].

The increased focus on achieving universal coverage of LLINs as a key malaria control intervention [4] for Mozambique and other countries is driven by experience [3] and modelling [5] showing that the indirect protective effects of bed nets at the community level can be greater than the direct protective effects of individual bed net use [5]. The bed nets’ repellent and insecticidal modes of action, together with the inherent dynamics of malaria transmission, can result in significant decreases in malaria transmission if a sufficient portion of all individuals at risk of malaria infection are consistently using the bed nets.

Despite the assumption that decreasing malaria transmission is dependent on achieving high coverage with bed nets, it is still unclear how to achieve universal coverage efficiently [6]. At issue is determining how many bed nets to procure and how to distribute them, with strategies ranging from a fixed number of bed nets per household regardless of size of household, one net for every two people, or one net for each sleeping space.

In place of any of the above three strategies, Mozambique piloted a novel universal coverage distribution model in Gaza Province in 2009. This distribution model involves an additional data-collection step during the ‘pre-census’ stage prior to distribution. Community leaders in the communities targeted for the campaign are requested to produce lists with the total number of households in their communities, the number of members in each household and their ages, sex and relationship with household head. These data are used, along with locally determined assumptions on sleeping patterns, to calculate an expected number of sleeping spaces. The household then receives a number of nets equal to the number of expected sleeping spaces. The use of the expected number of sleeping spaces differs from distribution strategies where data on the reported number of sleeping spaces are recorded during the pre-census stage [7].

This novel distribution model was employed in April 2010 in four districts in Sofala Province in central Mozambique [8]. Malaria parasitaemia prevalence in children under five in Sofala Province was measured to be 40% in the 2007 Malaria Indicator Survey [9]. The four districts participating in the distribution campaign had a total population of 218,537 distributed among 244 communities as determined during the pre-census. Community leaders conducted the household pre-census and enumerated the population, including recording the above-mentioned data on household composition. A total of 140,000 LLINs were then distributed according to calculations done by the distribution workers trained to calculate the number of sleeping spaces according to predetermined, standard sleeping pattern assumptions.

Two cross-sectional surveys were then conducted in the four districts, the first in May 2010, one month after the distribution, and the other 14 months after the campaign, in June and July of 2011. The surveys measured ownership, access and usage of the LLINS in randomly selected households, and also included collection and laboratory analysis of blood samples from selected individuals present in the surveyed households. The objective of these two surveys was to assess the coverage achieved by this distribution campaign, as well as to determine the impact on transmission, as measured by malaria parasitaemia and anaemia.

## Methods

### Study design and data collection

Two community-based, cross-sectional, household surveys were conducted after the 2010 universal coverage LLIN distribution campaign in four districts in Sofala Province: the first one in May 2010 and the second one in June and July 2011, one and 14 months after the campaign, respectively. The study protocol was approved by the Mozambican National Bioethics Committee and by the Hospital Clinic of Barcelona Bioethics Committee.

Households were selected using two-stage, cluster random sampling. First, 33 communities were selected using probability proportional to size across all four districts. From each selected community, 50 households were randomly selected to be visited. The sampling frames were derived from the predistribution census conducted in 2010. The same communities were used for both the 2010 and 2011 surveys, but sampling of households was done independently for each year. At the start of study activities in 2010, four communities that were selected in the initial sample were found to be inaccessible and infeasible to include in the study and were replaced with four nearby communities in the same districts that were more accessible, selected purposefully. One community that was visited in 2010 was not reachable in 2011. The study was powered to measure prevalence of malaria infection in children under five with a precision of 5%, assuming a prevalence of malaria infection of 50%. Assuming a design effect of 2, the minimum sample size was 768 children under five for each year.

Households were visited by trained survey teams and data were collected using standardised questionnaires. Geographic coordinates for each visited household were recorded and data on socio-economic status were collected. Visits also included collection of data on sleeping patterns (2010), direct observation of bed nets and sleeping spaces (both years), and recording of reported frequency of use of bed nets (both years). To record sleeping patterns, a roster of all household members was taken, and each sleeping space in the household was linked to the household members sleeping in that sleeping space. For both years, for each sleeping space, the interviewed household member was asked if there was a bed net designated (i.e. assigned) for the sleeping space, and the location of the bed net (hanging, stored, etc.) and the reported number of times per week that the bed net was used during the wet and dry season were recorded. The origin of each bed net was recorded, with campaign LLINs able to be identified because all campaign LLINs had been marked prior to the distribution campaign. Written informed consent was obtained from individual members of the household present during the visit, and consent was obtained from parents or guardians for children under 18 years of age.

In 2010, capillary blood specimens were obtained via finger-prick on present children under five years old in all selected households, present children between five and 14 years of age in half of households selected at random, and present adults in a quarter of households selected at random, using systematic sampling. In 2011, the sampling strategy was adjusted due to lower-than-expected sample size in 2010 and finger-pricks were performed on all present individuals in all selected households. All individuals tested were asked about bed net use the previous night. For children under five, haemoglobin concentration was measured using a HemoCue (AB Leo Diagnostics, Helsinborg, Sweden) portable device to assess anaemia, blood smears were prepared, a rapid diagnostic test (RDT) was performed, and a filter paper with three dried blood spots was collected. For sampled participants older than five years of age, a RDT for malaria was performed and three dried blood spots were collected. Results of the RDT were immediately communicated to study participants, and participants with positive RDTs were treated according to Mozambican national treatment guidelines. Children with haemoglobin <10 g/dL received a one month treatment of ferrous sulphate. Blood slides were Giemsa-stained in health facilities in the area and were later transported to be read for the presence of asexual *Plasmodium falciparum* parasites at the Manhiça Health Research Centre laboratories by two blinded technicians according to standard procedures. A third reading was performed if there was discrepancy between the first two. Paper forms were digitized using double data entry at the Manhiça Health Research Centre using OpenClinica, and data were analysed using R version 3.0.1 (R Core Team, Vienna, Austria).

### Definitions

To evaluate the coverage achieved during the campaign, a set of ownership, access and usage indicators (Table 1) was defined, including several based on the standard Roll Back Malaria Monitoring and Evaluation Reference Group (MERG) indicators [10].

**Table 1:** **Description of coverage indicators used in evaluation of long-lasting insecticidal net (LLIN) distribution campaign in Sofala Province, Mozambique**

Indicator	Unit of analysis	Definition
Ownership (campaign LLINs only)		
Proportion of households with at least one LLIN^a^	Household	Proportion of households receiving at least one campaign LLIN.
Access (campaign LLINs only)		
Proportion of households with sufficient LLINs	Household	Proportion of households receiving at least 1 campaign LLIN per sleeping space
Proportion of households with at least 1 LLIN for every 2 people^a^	Household	Proportion of households receiving at least 1 campaign LLIN per 2 people.
Proportion of sleeping spaces covered by LLIN	Sleeping space	Proportion of sleeping spaces with a campaign LLIN designated for the sleeping space
Proportion of population with access to LLIN within their household (actual)	Individual	Proportion of individuals sleeping in spaces with a campaign LLIN designated for the sleeping space
Proportion of population with access to an ITN within their household (estimated)^a^	Individual	Estimated proportion of individuals covered by campaign LLIN, where each LLIN is estimated to cover 2 individuals.
Usage (all bed nets)		
Proportion of sleeping spaces with a hung bed net	Sleeping space	Proportion of sleeping spaces for which a bed net was found hanging from the ceiling during household visit
Proportion of bed nets used ≥4 times a week during wet season	Sleeping space	Proportion of sleeping spaces for which a bed net was reported to be used ≥4 times a week during wet season
Proportion of bed nets used ≥4 times a week during dry season	Sleeping space	Proportion of sleeping spaces for which a bed net was reported to be used ≥4 times a week during dry season
Proportion of individuals sleeping under a bed net last night^a^	Individual	Proportion of individuals reporting having slept under a bed net during previous night.

^a^Based on Monitoring and Evaluation Reference Group indicator.

Achieved LLIN ownership was defined as the proportion of households that received at least one LLIN during the campaign. Access was evaluated at the household, sleeping space, and individual level. Access at the household level was defined as the proportion of households receiving sufficient campaign LLINs (at least one per sleeping space) and the MERG equivalent, the proportion of households receiving at least 1 campaign LLIN per two people. At the sleeping-space level, access was defined as the proportion of sleeping spaces for which a household had a campaign LLIN that was designated for that sleeping space. Finally, access at the individual level, the proportion of the population with access to a campaign LLIN within their household, was calculated as the proportion of individuals recorded as sleeping in a sleeping space for which a household had a campaign LLIN that was designated for that sleeping space (evaluated only for 2010). This last indicator was also estimated using the MERG method, calculating the total number of individuals covered per household as the number of campaign LLINs multiplied by 2 or the number of household members (whichever smaller) [11].

Four usage indicators were calculated: the proportion of all sleeping spaces that were observed to have a bed net located hanging above them; the proportion of sleeping spaces for which bed nets were reported to be used four or more times per week for the wet and dry season in the past year; and the proportion of individuals reporting having slept under a bed net the night before. Because of colinearity among the usage indicators (as assessed by Pearson’s correlation coefficient), a composite usage index for each household was also calculated, taking the first principal component of the four usage indicators outlined above.

Malaria infection was defined as a thick blood smear positive for any asexual *P. falciparum* parasitaemia, and anaemia as haemoglobin <11 g/dL. A composite socio-economic status (SES) index, scaled from 0 to 1, was calculated using principal components analysis of responses to questions regarding socio-economic conditions such as household ownership of goods, type of construction of house, level of education, literacy, and type of occupation [12].

### Analysis

Demographic characteristics of sampled households were tabulated. The point estimates and 95% confidence intervals of the ownership, access, and coverage indicators were calculated using the svyciprop command in the R survey package [13], adjusting for the two-stage cluster sampling, and calculating selection probabilities at both the community and household level. Similarly, the point estimates and 95% confidence intervals, as well as the design effect, were calculated for the proportion of children under five with blood slides positive for asexual *P. falciparum* parasites for 2010 and 2011.

Next, predictors of malaria parasitaemia and anaemia in 2011, one year after the distribution campaign, were investigated. Because LLINs are thought to influence malaria risk at multiple scales, including both the household and community level, new variables representing community-level LLIN access and LLIN usage were constructed. For each community, the average community LLIN access (the proportion of all sleeping spaces in a community that had a campaign LLIN designated for the sleeping space) and average community usage (the composite usage index averaged across all sleeping space in a community) were calculated. To accurately separate the community and household-level effects, these community averages were subtracted from the household-level access (the proportion of sleeping spaces in a household that had a campaign LLIN designated for the sleeping space) and household-level usage (the composite usage index averaged across all sleeping space in a community) [14].

Using multivariate analysis, individual-level malaria parasitaemia and anaemia results in 2011 were regressed against household LLIN access, household LLIN usage, community LLIN access, and community LLIN usage as possible predictors, and age, SES, and community-level malaria prevalence and anaemia prevalence in 2010 as confounders. Both usage and access variables were included to adjust for the effect of differential access on usage. An age-squared term was included as the relationship between risk of malaria infection and anaemia and age was found to be non-linear. Regressions were performed using R function svyglm in the survey package, using the svydesign function to specify weights as calculated above [R scripts available upon request].

## Results

A total of 1,362 and 1,330 households were surveyed in 2010 and 2011, respectively. Household size ranged from one to 17 persons, with roughly half of the surveyed households having fewer than five household members; most of the surveyed households were found in the most populous district, Gorongosa (Table 2). The members of the sampled households represented a total population of 6,555 in 2010 and 6,389 in 2011.

**Table 2:** **Characteristics of households visited in evaluation of universal coverage long-lasting insecticidal net distribution campaign in Sofala Province, Mozambique**

	1 month after campaign	14 months after campaign
Number of households	1,362	1,330
Household size (%)		
<5	647 (47)	677 (51)
5-9	626 (46)	566 (43)
>9	89 (7)	87 (7)
District (%)		
Nhamatanda	235 (17)	254 (19)
Gorongosa	698 (51)	672 (51)
Cheringoma	262 (19)	235 (18)
Muanza	167 (12)	169 (13)
Number of sleeping spaces examined	3,322	2,895
Number of campaign bed nets examined	2,575	2,442
Number of household members	6,555	6,389

Post-campaign LLIN ownership was high. As assessed one month after the campaign, 98% (95% CI: 97-99%) of households had received at least one campaign LLIN; in the one-year follow-up survey, 93% (95% CI: 91-95%) of households reported having received at least one LLIN during the campaign (Table 3). The proportion of all households having received a sufficient number of LLINs during the campaign, greater or equal to the number of sleeping spaces, was 85% (95% CI: 81-88%) in 2010 and 86% (95% CI: 83-89%) in 2011. The household-level access estimated using the MERG 2 person per LLIN access indicator was lower, measured at 67% (95% CI: 64-70%) in 2010 and 62% (95% CI: 58-66%) in 2011. The discrepancy in the two house-level access indicators was because the 2 person per sleeping space assumption did not hold in most households (see Additional file 1: Table S1). At the sleeping-space level, 81% (95% CI: 77-85%) of sleeping spaces in 2010 and 81% (95% CI: 75-86%) of sleeping spaces in 2011 had a campaign LLIN designated for them. Finally, at the individual-level, 85% (95% CI: 81-88%) of individuals in 2010 slept in a sleeping space that had a campaign LLIN designated for them. The MERG individual-level access estimate was 88% (95% CI: 86-89%) in 2010 and 81% (95% CI: 78-84%) in 2011.

**Table 3:** **Coverage indicators following universal coverage long-lasting insecticidal net (LLIN) distribution campaign in Sofala Province, Mozambique**

	% (95% CI)	
	1 month after campaign	14 months after campaign
Ownership (campaign LLINs only)		
Proportion of households with at least one LLIN	98 (97–99)	93 (91–95)
Access (campaign LLINs only)		
Proportion of households with sufficient LLINs	85 (81–88)	86 (83–89)
Proportion of households with at least 1 LLIN for every 2 people	67 (64–70)	62 (58–66)
Proportion of sleeping spaces covered by LLIN	81 (77–85)	81 (75–86)
Proportion of population with access to LLIN within their household (actual)	85 (81–88)	^b^
Proportion of population with access to an ITN within their household (estimated)	88 (86–89)	81 (78–84)
Usage (all bed nets)		
Proportion of sleeping spaces with a hung bed net	61 (56–66)	65 (57–72)
Proportion of bed nets used ≥4 times a week during wet season^a^	^b^	65 (56–74)
Proportion of bed nets used ≥4 times a week during dry season^a^	^b^	60 (52–68)
Proportion of individuals sleeping under a bed net last night		
<5 years	94 (91–96)	79 (74–83)
5–14 years	88 (82–93)	74 (67–80)
>14 years	88 (82–92)	76 (71–81)

^a^In preceding year.

^b^Not assessed.

In the year following the campaign, 65% (95% CI: 56-74%) of sleeping spaces in the wet season and 60% (95% CI: 52-68%) of sleeping spaces in the dry season were covered by a bed net reportedly used more than four times a week. This is lower than the proportion of sleeping spaces covered by campaign LLINs because not all bed nets were reported to be used four or more times per week; only 78% (95% CI: 67-86%) of bed nets were reported to have been used four or more times per week during the wet season, falling to 71% (95% CI: 62-80%) in the dry season.

The observation that even when bed nets are available, they are not always used is reinforced by data on the location of bed nets. Only 80% (95% CI: 74-84%) of bed nets in 2010 and 79% (95% CI: 73-84%) of bed nets in 2011 were found to be hanging over a sleeping space. In combination with the incomplete coverage achieved this resulted in 61% (95% CI: 56-66%) of sleeping spaces in 2010 and 65% (95% CI: 57-72%) of sleeping spaces in 2011 being observed to have hanging bed nets.

Usage, as measured as the proportion of individuals sleeping under a bed net the previous night, declined from 2010 to 2011. In 2010, 94% (95% CI: 91-96%) of children under five were reported to have slept under a bed net the night before; in 2011, this decreased significantly to 79% (95% CI: 74-83%). Other age classes had overall lower usage and showed a similar decline in reported use (Table 3).

Blood smears for 602 and 624 children under five were read in 2010 and 2011, respectively. The estimated prevalence of malaria parasitaemia was 47% (95% CI: 40-54%) one month after baseline and 36% (95% CI: 27-45%) 14 months after baseline. The design effect for this measure was calculated to be 3.4 for 2010 and 5.5 in 2011. The prevalence of anaemia, defined as haemoglobin <11 g/dL, in children under five was 72% (95% CI: 67-77%) one month after baseline and 68% (95% CI: 62-73%) 14 months after baseline.

In the multivariate analyses (Table 4), community-level usage of bed nets was found to be significantly associated with a reduction in risk for malaria infection (aOR: 0.21, 95% CI: 0.065-0.69) and risk for anaemia (aOR: 0.043, 95% CI: 0.0091-0.2) in children under five. Household-level usage of bed nets, community-level access to bed nets, and household-level access to bed nets were not significantly associated with risk for malaria infection or anaemia.

**Table 4:** **Predictors of malaria infection and anaemia in children under five, 14 months after a universal coverage bed net distribution campaign in Sofala Province, Mozambique, multivariate analysis**

	Adjusted odds ratio (95% CI)	
Risk factor	Outcome: malaria infection	Outcome: anaemia
Community malaria prevalence in 2010^a^	**22 (5.3-93)**	**-**
Community anaemia prevalence in 2010^a^	**-**	2.3 (0.83-6.2)
SES index^b^	0.46 (0.11-1.9)	0.69 (0.19-2.5)
Male sex	0.67 (0.44-1)	0.74 (0.5-1.1)
Age in years	3.3 (1.7-6.3)	0.62 (0.37-1)
Age^2^ in years	**0.85 (0.75-0.95)**	1 (0.93-1.1)
Community bed net usage index^b^	**0.21 (0.065-0.69)**	**0.043 (0.0091-0.2)**
Household bed net usage index^b^	0.93 (0.46-1.9)	0.52 (0.23-1.2)
Community bed net access^c^	3.7 (0.44-32)	2.2 (0.19-25)
Household bed net access^c^	1.4 (0.62-3.3)	2.7 (0.93-7.7)

^a^1 month after the distribution campaign.

^b^Index normalized to be between 0 (minimal value) and 1 (maximal value).

^c^Proportion of individuals having access to a campaign LLIN.

Boldface indicates statistical significance.

## Discussion

By observing all sleeping spaces during household visits and recording who slept in which sleeping space, it was possible to precisely characterize the access to campaign LLINs following the campaign. The proportion of households receiving enough LLINs to cover all sleeping spaces (85%), of sleeping spaces covered by campaign LLINs (81%), and of individuals sleeping in sleeping spaces with campaign LLINs designated for them (85%) were able to be calculated. The high access indicators measured after the campaign suggest that campaigns using the novel distribution model introduced in Mozambique are able to achieve high coverage.

However, it needs to be noted that about a fifth of sleeping spaces were not covered by campaign LLINs. Moreover, even in sleeping spaces covered by bed nets, one-fifth of the bed nets were not hung and one-fifth of the bed nets were reported to be used less than four times per week. Together, this resulted in incomplete protection of the population, with only 65% of sleeping spaces observed to have a hanging bed net, and 65% of sleeping spaces covered by a bed net reported to be used more than four times per week during the wet season (falling to 60% in the dry season).

An association between this usage and reduced malaria transmission in the entire study area was not detected. Although the results show an 11 percentage-point fall in parasitaemia prevalence from the baseline to roughly one year after the universal coverage distribution campaign, the confidence intervals for both years overlap. A larger than expected design effect (3.4 in the first year and 5.5 and in the second year, compared to an expected design effect of 2) meant that the study was substantially underpowered. Because of the highly non-linear relationship between transmission intensity and prevalence of parasitaemia [15], future studies aimed at measuring impact might consider larger sample sizes or alternate indicators.

However, it was possible to document a potential impact in the subset of the population using bed nets, with community-level usage of bed nets significantly associated with a reduction in malaria risk one year after the campaign, assuming no changes in malaria treatment practices during the evaluation period. Previous studies analysing data from cluster randomized trials, typically comparing intervention villages receiving bed nets and control villages, have confirmed that the protective effect of bed nets and curtains functions on a scale larger than the individual. The community-level effect is reflected in both entomological indicators, such as mosquito abundance, sporozoite carriage rate and entomological inoculation rate [16, 17], as well as health outcomes such as mortality and hospital admissions [3, 18, 19]. In these cluster-randomized trials, the protective effects of bed nets have been found to be measurable up to several hundred metres from intervention villages.

However, in a non-trial setting, these spatial, community effects have been harder to measure [20]. Measuring this community-level effect during universal coverage distribution campaigns is difficult because bed net ownership and access should be uniformly high after universal coverage distribution campaigns, so there is often too little variation in the variable of exposure. The analysis presented here, however, shows that the community-level protective effect of bed nets is still detectable in an area with uniformly high coverage. Importantly, the effect is only measurable if one considers usage of bed nets instead of ownership as a predictor. There is enough variation in household usage of bed nets even in an area experiencing a universal coverage distribution campaign to be able to measure the indirect protective effects of bed nets.

There were several limitations to the methodology used in the study. The sampling methodology could have resulted in an overestimate of the association between the campaign and the prevalence of parasitaemia. Because the sampling frame was derived from lists used to distribute LLINs, new households and households that were not captured during the census and did not participate in the distribution campaign were excluded. As a result, the coverage, access, and usage indicators were likely overestimated. Inability to visit certain villages due to inaccessibility also likely led to overestimation of measures of coverage and impact, as the most remote villages are least likely to have been well served by the campaign. The timing of the surveys could also have biased the results, with the fact that the baseline was measured one month after the campaign likely resulting in underestimation of the impact, and the fact the second year was conducted later in the transmission season than the first year possibly causing overestimation of the impact. The results showing that community-level use of LLINs is the best predictor of reduction in individual malaria risk, however, are not subject to these constraints. Finally, control areas that had not benefited from the universal coverage campaign could not be included in the surveys, preventing formal testing of the causality of the intervention.

The importance of frequency of bed net usage, compared to ownership or access reinforces the need to measure this indicator when evaluating bed net distribution campaigns and malaria control programme performance as well as assessing malaria risk. More evaluations are needed to identify what aspects of distribution models can influence future bed net usage and result in more profound reductions in malaria risk at both the individual and community level.

## Electronic supplementary material

Additional file 1: Table S1: Cross-tabulation of number of beds in household versus the total number of household members, from 2010 and 2011 evaluation of universal coverage distribution campaign in Sofala Province, Mozambique. (PDF 31 KB)
