# Supplementary material for: Evaluation of a universal coverage bed net distribution campaign in four districts in Sofala Province, Mozambique
Source: Malar J. 2014 Nov 5;13:427. doi: 10.1186/1475-2875-13-427 (PMC4232630; doi:10.1186/1475-2875-13-427)
Supplement: Supplementary file 1 — Additional file 1: Table S1: Cross-tabulation of number of beds in household versus the total number of household members, from 2010 and 2011 evaluation of universal coverage distribution campaign in Sofala Province, Mozambique. (PDF 31 KB) [file 12936_2014_3585_MOESM1_ESM.pdf]

Table S1. Cross-tabulation of number of beds in household versus the total number of household members, from 2010 and 2011 evaluationof universal coverage distribution campaign in Sofala Province, Mozambique

Number of Household Members	Number of Beds in Household: 1	2	3	4	5	6	7	8
1	180 (99)	2 (1.1)	0	0	0	0	0	0
2	217 (72)	81 (27)	0	2 (<1)	0	0	0	0
3	179 (46)	171 (44)	35 (9.1)	1 (<1)	0	0	0	0
4	88 (20)	250 (56)	96 (21)	16 (3.6)	0	0	0	0
5	19 (4.8)	211 (53)	136 (34)	29 (7.3)	0	0	0	0
6	10 (3)	115 (35)	157 (48)	34 (10)	12 (3.7)	0	0	0
7	5 (1.9)	66 (25)	133 (51)	45 (17)	13 (5)	0	0	0
8	4 (2)	15 (7.4)	94 (46)	61 (30)	24 (12)	4 (2)	0	1 (<1)
9	0	4 (4.8)	36 (43)	27 (32)	13 (15)	4 (4.8)	0	0
10	0	3 (5.8)	11 (21)	16 (31)	16 (31)	2 (3.8)	1 (1.9)	3 (5.8)
11	0	1 (6.7)	1 (6.7)	4 (27)	3 (20)	3 (20)	2 (13)	1 (6.7)
12	0	0	1 (9.1)	0	6 (55)	0	2 (18)	2 (18)
13	0	0	0	0	2 (40)	3 (60)	0	0
14	0	0	0	2 (100)	0	0	0	0
15	0	0	1 (100)	0	0	0	0	0
16	0	0	0	0	0	2 (100)	0	0
17	0	0	0	0	0	1 (50)	0	1 (50)

The number in each cell indicates count of households. The numbers in parentheses indicate row percentages. Gray shading indicates the 2 people per sleeping space assumption underlying the Roll Back Malaria Monitoring and Evaluation Reference Group (MERG) access indicators. Overall, the 2 people per sleeping space assumption was met in 47.7% of households, with 39.6% of households having fewer sleeping spaces than expected, and 13.7% of households have more sleeping spaces than expected.
